# Hybrid Three-Mode Correlation and Squeezing in a Pr^3+^:YSO Crystal

**DOI:** 10.1038/s41598-017-01860-8

**Published:** 2017-05-11

**Authors:** Zongchen Liu, Irfan Ahmed, Garuma Abdisa, Zihai Jiang, Shuwei Fan, Hongxing Wang, Yanpeng Zhang

**Affiliations:** 1 Shaanxi Key Lab of Information Photonic Technique, Xi’an Jiaotong University, Xi’an, 710049 China; 2 Key Laboratory for Physical Electronics and Devices of the Ministry of Education, Xi’an Jiaotong University, Xi’an, 710049 China; 3 Institute of Wide Bandgap Semiconductors, Xi’an Jiaotong University, Xi’an, 710049 China; 4 Department of Physics and Materials Science, City University of Hong Kong, Kowloon Tong, Hong Kong SAR; 5 Electrical Engineering Department, Sukkur IBA, Sukkur, 65200 Sindh Pakistan

## Abstract

We report the generation of three-mode hybrid intensity-noise correlation and intensity-difference squeezing of spontaneous parametric four-wave mixing (SP-FWM) and fourth-order fluorescence (FL) signals in the heteronuclear-like (three-level Λ-type) molecular structure of a Pr^3+^:Y_2_SiO_5_ (Pr^3+^:YSO) crystal using the nonlinear cross-Kerr effect under a polarized dressing effect. In the semi-classical view of a Kerr nonlinear medium, the amplitude of two-mode hybrid correlations of this kind is subject to a limit determined by the hybrid maximally entangled state. Whereas the degree of correlation and squeezing is determined by the dressing effects of the input laser fields participating in the SP-FWM process. We also find that the variations in magnitude of three-mode hybrid intensity-noise correlation and intensity-difference squeezing are consistent with nonlinear cross-Kerr processes. Such a three-mode hybrid signal may have potential applications in long-distance communication, dense coding, all-optical communication and quantum storage on photonic chips.

## Introduction

The generation of entangled photons is of particular interest in fundamental studies of quantum mechanics and has potential applications in information processing and long-distance communications. In recent decades, scientists have created various photon states, such as coherent states^1^ and single-photon states^2^, for various applications. In addition, classical^3^ and non-classical^4^ photon correlations have been achieved under various experimental conditions. Recently, hybrid entangled states^5^, which involve entanglement between classical and non-classical photons, have been identified as useful resources for long-distance communications^6^ and optical quantum information processing^7, 8^, as such a hybrid state acts as a new type of qubit^9^. This approach has received considerable attention because of its incorporation of the advantages of both classical and non-classical photon states, especially for loophole-free Bell inequality tests using inefficient detectors^10^, near-deterministic quantum teleportation using linear optics and hybrid qubits^8^ and information transfer between different types of qubits^11^. Thus, the generation of hybrid entangled states is crucial. The most widely used technique for the generation of entangled photon pairs is spontaneous parametric downconversion (SPDC) (*χ* ^(2)^ nonlinearity) using a nonlinear crystal^12^. However, the photons generated via SPDC have a very wide bandwidth (~THz), making it difficult to realize effective coupling between photons and atoms. Such coupling is essential for long-distance communications^13^. The filter-driven four-wave mixing (FWM) effect (*χ* ^(3)^ nonlinearity) can induce a high repetition rate of an ultrafast fibre laser^14^, and FWM can induce the energy transfer of different waves with a self-stabilizing function, as first reported by Liu *et al*.^15^, and can be used as a basis for the development of multi-wavelength fibre lasers^16–18^. Moreover, spontaneous parametric FWM (SP-FWM) using a rare-earth-ion-doped Pr^3+^:Y_2_SiO_5_ (Pr^3+^:YSO) crystal preserves “atom-like” properties, with a long coherence time (0.1–1 s) and a narrow spectral width (~MHz)^7, 19^. In such a nonlinear crystal, the dopant cations are localized at different vacancy sites arising from dipole-dipole interactions, giving rise to different energy-level configurations^20^. Therefore, when multiple Zeeman energy levels are involved in the atomic systems of the nonlinear crystal, the effects of dressing fields can be used to control the process^21, 22^. Moreover, an SP-FWM process generates pairs of Stokes and anti-Stokes photons accompanied by a simultaneously generated fourth-order fluorescence (FL) signal. Recently, hybrid-cascaded sources from the SP-FWM and spontaneous Raman scattering processes in atomic ensembles using nonlinear processes of different orders have been studied^23^. Intensity-noise correlated and intensity-difference squeezed beams have important applications in quantum metrology and gravitational-wave detection. In addition, Jietai Jing’s group has experimentally demonstrated that increasing the number of modes of entanglement enhances the degree of correlation and squeezing^19^, and this phenomenon also shows potential for application to increase the information rate in dense coding^24^.

Motivated by these achievements, two-mode intensity-noise correlation and intensity-difference squeezing of SP-FWM and fourth-order FL signals have been investigated^25^. In this paper, we focus on a hybrid state with three-mode intensity-noise correlation and intensity-difference squeezing of SP-FWM and hybrid FL signals in a Pr:YSO crystal using the nonlinear cross-Kerr effect under polarized dressing fields as Kerr non linearity is directly proportional to the square of the electric field. We investigated the differences between the three-mode hybrid correlation and squeezing effects induced by the cross-Kerr effect under linearly and circularly polarized dressing fields. We found that the degree of three-mode hybrid correlation and squeezing induced by the cross-Kerr effect decreases when the dressing field is changed from a linear polarization to a circular polarization. We also found that the FL signal interacts with the Stokes/anti-Stokes photon pairs and that the degree of hybrid intensity-noise correlation and intensity-difference squeezing can be controlled by controlling the emission of the hybrid FL signal. Our results obtained in this atom-like medium indicate several obvious advantages, such as an improved integration ability compared to atomic vapours and a long coherence time compared to other traditional solid materials. These achievements also have potential applications in all-optical communication and quantum storage on photonic chips.

## Results and Analysis

### Experimental setup

**Figure 1:**
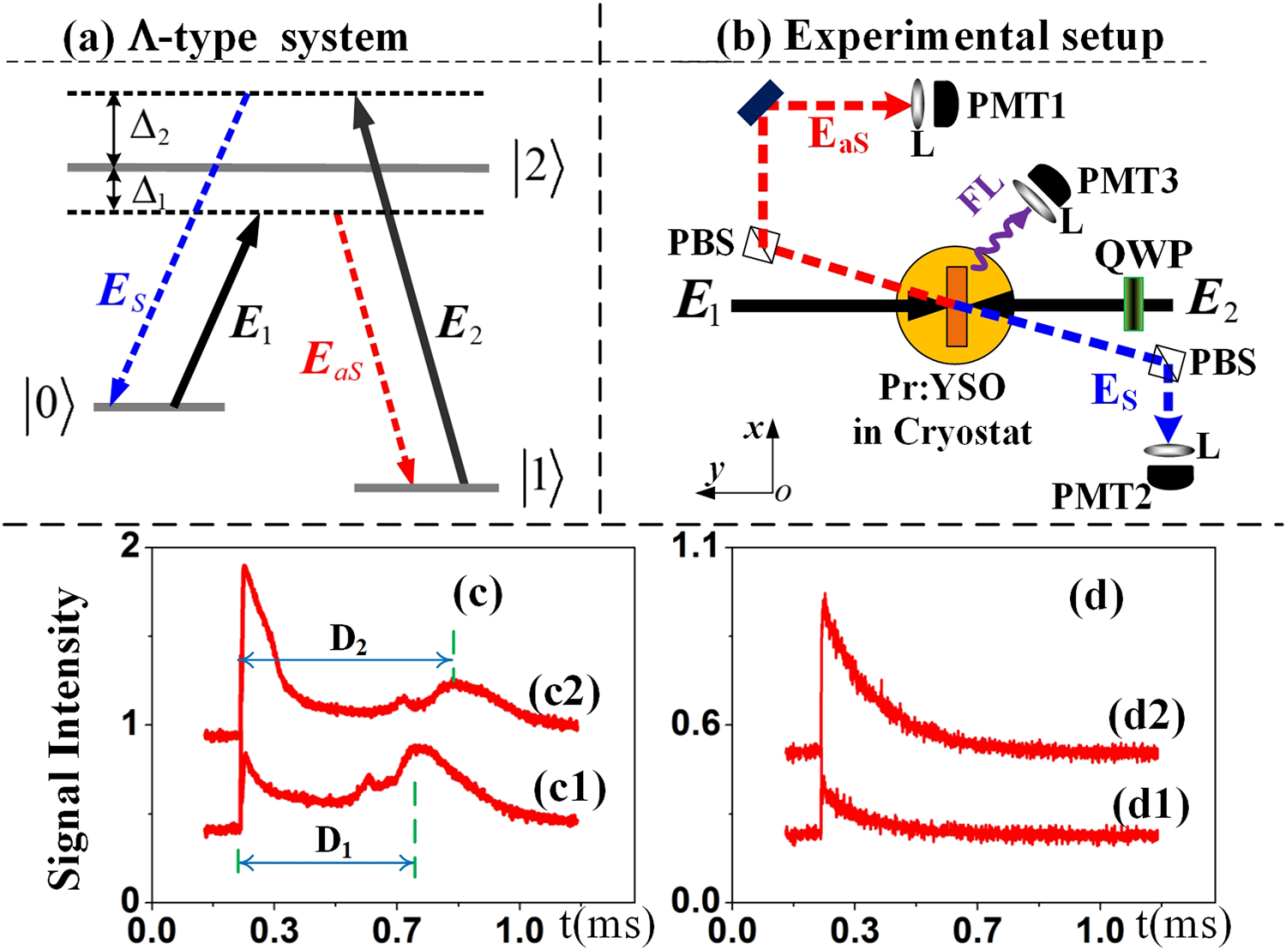
(**a**) The Λ-type atomic system in a Pr^3+^:YSO crystal and the laser coupling configurations. (**b**) The experimental setup, where Δ_1_ and Δ_2_ are the frequency detunings of the fields *E*_1_ and *E*_2_, respectively; PMT denotes a photomultiplier tube; L denotes a lens; PBS denotes a polarizing beam splitter; and QWP denotes a quarter-wave plate. (**c**) The intensity of the hybrid FL+SP-FWM signal in the time domain as the power increases from 2 mW in (c1) to 6 mW in (c2). (**d**) The intensity of the pure SP-FWM signal in the time domain for the same power increment as in (c1) and (c2).

**Figure 2:**
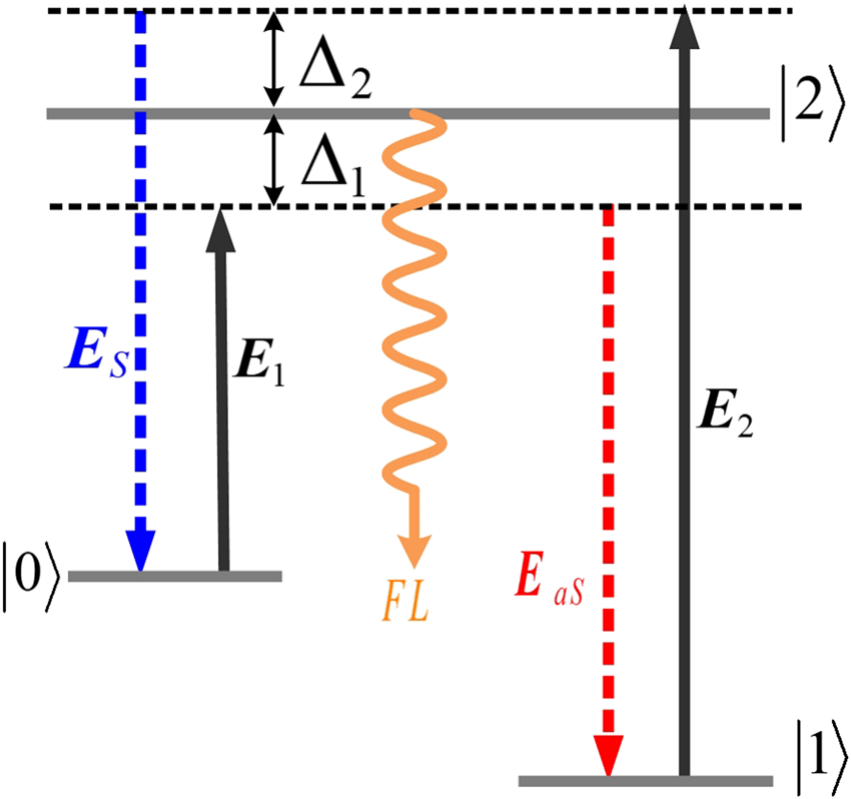
Level scheme for the cross-Kerr effect. *E*_1_ and *E*_2_ are the pumping and dressing fields, respectively. The weak Stokes field (*E*_*S*_), anti-Stokes field (*E*_*aS*_) and fluorescence field (FL) constitute the hybrid signal.

A rare-earth-ion-doped (0.05% Pr^3+^) 3-mm YSO (Y_2_SiO_5_) crystal was used in the experiment. Coupling can occur between Pr^3+^ ions localized at different cation vacancies as a result of induced dipole-dipole interactions; consequently, one can treat two such ions as a heteronuclear-like molecule. Therefore, we can construct a Λ-type three-level system (as shown in Fig. 1(a)) by coupling laser fields to the corresponding atomic transition levels, as shown in Fig. 1(b). Two tunable dye lasers (with a narrow scan of 0.04 cm^−1^ in linewidth) pumped by an injection-locked single-mode Nd:YAG laser and a Continuum Powerlite DLS 9010 with a 10 Hz repetition rate and a 5 ns pulse width were used to generate the pumping field *E*_1_(*ω*_1_, Δ_1_) and the dressing field *E*_2_(*ω*_2_, Δ_2_) with frequency detunings of ${{\rm{\Delta }}}_{i}={{\rm{\Omega }}}_{mn}-{\omega }_{i}$, where ${{\rm{\Omega }}}_{mn}$ is the corresponding atomic transition frequency between levels |*m*〉 and |*n*〉 and *ω*_*i*_ (*i* = 1, 2) is the laser frequency. The pumping field *E*_1_ (for which the power, polarization and detuning were fixed at 5 mW, linear polarization and 0 GHz, respectively) and the dressing field *E*_2_ were coupled into the crystal in opposite directions. A quarter-wave plate (QWP) was used to control the polarization state of the dressing field *E*_2_, and the power *P*_2_ and detuning Δ_2_ of the dressing field *E*_2_ could be modified by changing the parameters of the dye lasers. The Stokes and anti-Stokes photons were produced under the phase-matching condition **k**_**1**_ + **k**_**2**_ = **k**_**S**_ + **k**_**aS**_ and the energy conservation condition *ω*_1_ + *ω*_2_ = *ω*_*S*_ + *ω*_*aS*_, where **k** and *ω* denote the wave vectors and transition frequencies, respectively, of the pumping field, the dressing field and the generated photon pairs. Meanwhile, the hybrid photons were obtained via a Kerr nonlinear interaction under the influence of the higher photonic flux of the pump beam *E*_1_(*ω*_1_, Δ_1_), which passed through the nonlinear crystal as shown in Fig. 1(b). Two photomultiplier tubes (PMT1 and PMT2) were arranged as shown in Fig. 1(b) to detect the Stokes and anti-Stokes signals reflected by PBSs. The FL signals were simultaneously detected by PMT3 with a fast boxcar gated integrator; as shown in Fig. (2), these signals accompany the SP-FWM process under the cross-Kerr effect of *E*_1_ (pumping field) and *E*_2_ (dressing field). The weak Stokes field (*E*_*S*_), anti-Stokes field (*E*_*aS*_) and fluorescence field (FL) constitute the hybrid signal, whose propagation can be described as evolution under the Heisenberg picture^26, 27^; see the Methods section for further details.

### Results and discussion

Figure 1(c1) and (c2) show the intensity of the FL+SP-FWM signal in the time domain as the power of *E*_2_ increases from 2 mW (shown in (c1)) to 6 mW (in (c2)) when the polarization state of *E*_2_ remains fixed at 0° (linear polarization), the detuning of *E*_2_ is set to Δ_2_ = 0 GHz, and the power of *E*_1_ (*P*_1_) is held fixed. Figure 1(d1) and (d2) show the intensity of the pure SP-FWM signal in the time domain (obtained under the phase-matching condition for the SP-FWM process) under the same conditions as in Fig. 1(c). The total intensity of the hybrid SP-FWM+FL signal can be written as $\rho ={\rho }_{FL}^{(4)}+{\rho }_{SP-FWM}^{(3)}$, where ${\rho }_{SP-FWM}^{(3)}={\rho }_{20(S)}^{(3)}+{\rho }_{20(aS)}^{(3)}$ and ${\rho }_{FL}^{(4)}={\rho }_{11(FL)}^{(4)}$. Because of the dressing-induced competition effect between the FL and SP-FWM signals, the delay time between them increases^28^ with increasing power, as shown in Fig. 1(c1,c2). The delay times are indicated by the splitting distances (in units of time), labelled as *D*_1_ and *D*_2_, between the SP-FWM signal (left peak) and the FL signal (right peak) in Fig. 1(c). However, at a sufficiently high power, the FL signal is suppressed and the SP-FWM signal is enhanced because of the dressing-induced competition effect, as shown in Fig. 1(c1)–(c2) and 1(d1)–(d2); these findings agree with those of ref. 29. Thus, the FL signal is more sensitive to dressing than is the SP-FWM signal^30, 31^. We can make use of these properties of the FL signal to selectively monitor the input dressing laser’s polarization, power and frequency detuning in a Λ-type system to control the degree of hybrid three-mode intensity-noise correlation and intensity-difference squeezing.

**Figure 3:**
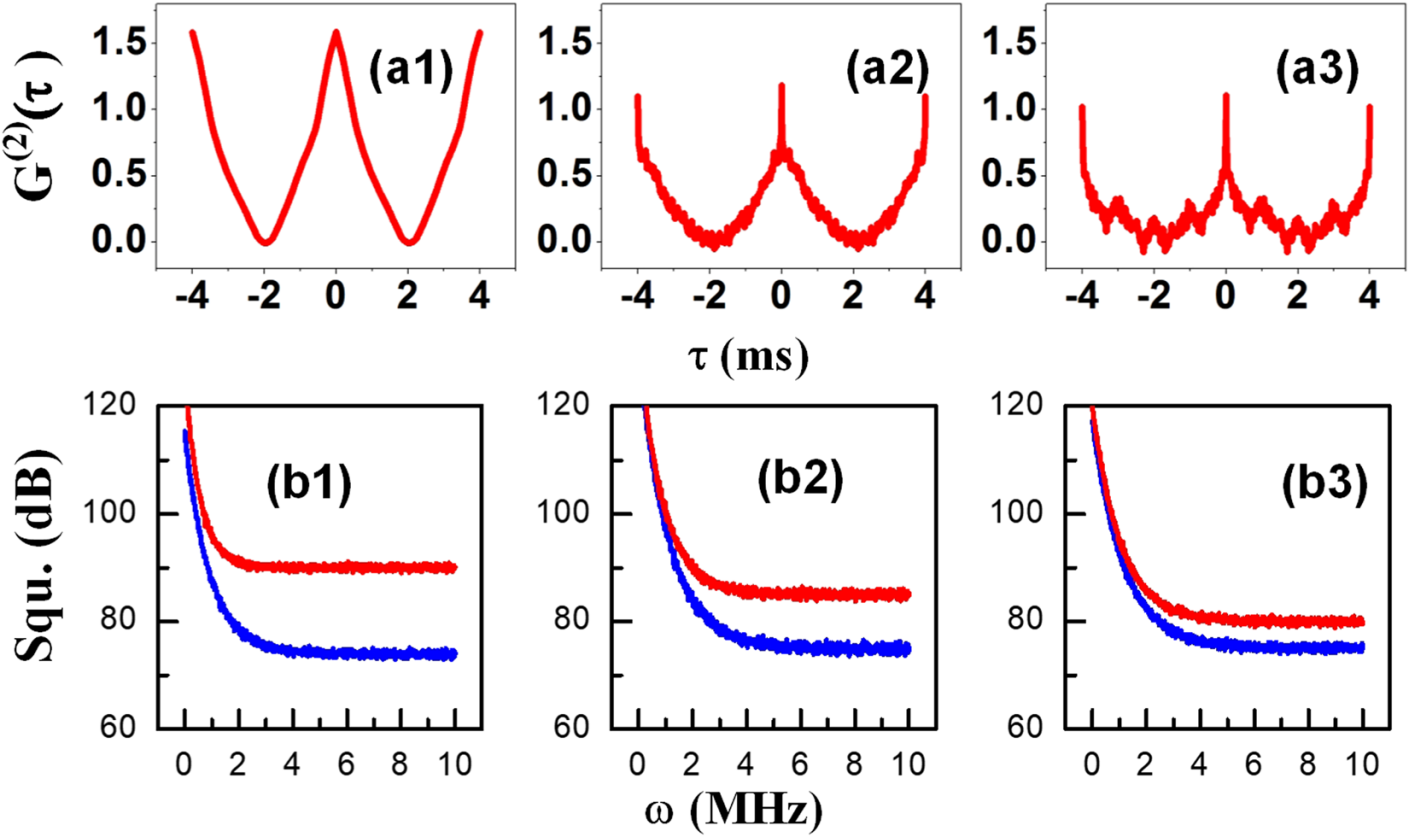
(**a1–a3**) Show two-mode intensity-noise correlations. (**a1–a3**) Show the intensity-noise correlations of the Stokes/anti-Stokes, Stokes/FL and anti-Stokes/FL signal pairs, respectively. **(b1–b3**) Show the intensity-difference squeezing results corresponding to (**a1–a3**), respectively.

Figure 3 shows the experimental results obtained for two-mode intensity-noise correlation and intensity-difference squeezing at the high power level of 6 mW represented in Fig. 1(c2), with the polarization of *E*_2_ fixed at 0° and the detuning of *E*_2_ set to Δ_2_ = 0 GHz. Under the cross-Kerr effect with the dressing field *E*_2_ in the linearly polarized state, Eqs (5) and (6) change to ${\rho }_{21(aS-FL)}^{(3)}=-i{G}_{aS}{c}_{x}^{2}{|{G}_{2}|}^{2}/{({{\rm{\Gamma }}}_{21}+i{{\rm{\Delta }}}_{1})}^{2}({{\rm{\Gamma }}}_{11}+i{{\rm{\Delta }}}_{1})$ and ${\rho }_{20(FL-aS)}^{(3)}=-i{G}_{S}{c}_{x}^{2}{|{G}_{2}|}^{2}/{{\rm{\Gamma }}}_{20}^{2}{{\rm{\Gamma }}}_{10}$, respectively.

The two-mode intensity-noise correlation between the Stokes and anti-Stokes signals (Fig. 3(a1)) can be obtained by substituting the intensity-noise signals detected by PMT1 and PMT2 into Eq. (17). Meanwhile, the intensity-noise correlations between the Stokes and FL signals (Fig. 3(a2)) and between the anti-Stokes and FL signals (Fig. 3(a3)) can be obtained by substituting the corresponding detected intensities into Eq. (19). Similarly, by substituting the detected intensity data into Eq. (18), we can also obtain the intensity difference ${\delta }^{2}({\hat{I}}_{S}-{\hat{I}}_{aS})$ (blue curve in Fig. 3(b1)) and the noise sum ${\delta }^{2}({\hat{I}}_{S}+{\hat{I}}_{aS})$ (red curve in Fig. 3(b1)) versus *ω* (analysis frequency) between the Stokes and anti-Stokes signals. Finally, the intensity differences ${\delta }^{2}({\hat{I}}_{S/aS}-{\hat{I}}_{FL})$ and the noise sums ${\delta }^{2}({\hat{I}}_{S/aS}+{\hat{I}}_{FL})$ versus *ω* between the Stokes and FL signals (Fig. 3(b2)) and between the anti-Stokes and FL signals (Fig. 3(b3)) can be obtained by substituting the corresponding detected intensity data into Eq. (20).

Figure 3(a1) shows the intensity-noise correlation between the Stokes and anti-Stokes signals from the SP-FWM process. A high correlation is observed between these two coherent signals, indicating that the two signals represent strongly correlated^32, 33^ and non-classical states. Furthermore, the degree of correlation (the height of the correlation curve) is approximately 1.5 at *τ* = 0, which corresponds to ${G}^{2}(\tau )\approx a\gg 1$; consequently, the output correlation can be treated as indicating a maximally entangled state, as the Stokes and anti-Stokes signals are determined by one another through four-wave mixing. The Stokes and anti-Stokes photons are produced under the phase-matching condition and the energy conservation condition. Hence, the Stokes and anti-Stokes signals are determined by each other, and the degree of correlation in the SP-FWM process is large.

By contrast, Fig. 3(a2) and (a3) show the correlation results for the Stokes/SP-FWM+FL and anti-Stokes/SP-FWM+FL signals, respectively. Here, the hybrid signal (SP-FWM+FL) is obtained via a cross-Kerr nonlinear interaction as shown in Fig. 2, and its propagation is defined as in Eqs (1) and (2), under a non-phase-matching and non-energy-conserving condition between the pumping field and the FL signal. Consequently, the degrees of correlation based on the cross-Kerr nonlinear processes are approximately 1.25 and 1.1, smaller than that for the pure SP-FWM process. As the SP-FWM+FL signal considered in these correlations is a hybrid signal, one can treat this as a hybrid entangled state $|\psi (a)\rangle =(|0\rangle |a\rangle +|1\rangle |-a\rangle )/\sqrt{2}$, where |0〉 and |1〉 represent Stokes/anti-Stokes and hybrid (SP-FMW+FL) photons, respectively. The correlation values obtained in this case are slightly lower than those for the maximally entangled Stokes and anti-Stokes states, which are obtained through a coherent process. This result is attributed to the mixture of the SP-FWM and FL signals under the cross-phase modulation of the Kerr interaction, which results in a larger decay rate of the SP-FWM signal compared to the FL signal. The squeezing between the intensity difference and noise sum of the Stokes/anti-Stokes signals (−15.19 dB) is larger than that of both the Stokes/FL signals (−8.92 dB) and the anti-Stokes/FL signals (−9.98 dB), as shown in Fig. 3(b1–b3), consistent with the correlations shown in Fig. 3(a1–a3).

In other words, because the Stokes and anti-Stokes photons are produced under conditions of phase matching and energy conservation, the Stokes and anti-Stokes signals are determined by each other, so the degree of correlation in the SP-FWM process is large. By contrast, there is no phase matching or energy conservation between the pumping field and the FL signal, so although the hybrid Stokes/SP-FWM+FL and anti-Stokes/SP-FWM+FL signals affect each other, their degrees of correlation via the cross-Kerr processes are smaller than that of the SP-FWM process.

Figure 3(a) also depicts the shape of the correlation function. A broad correlation peak is obtained from the coherent process, with a linewidth determined by the atomic coherence time. Thus, the SP-FWM signals from the coherent process decay rapidly, and the correlation shape is defined as ${A}_{{S}_{2}/a{S}_{2}}={R}_{1}{|{A}_{1}|}^{2}[{e}^{-(2{{\rm{\Gamma }}}^{-}+\zeta )|\tau |}+{e}^{-(2{{\rm{\Gamma }}}^{-}+\zeta )|\tau |}-2\,\cos \,({{\rm{\Omega }}}_{e}|\tau |){e}^{-({{\rm{\Gamma }}}^{+}+{{\rm{\Gamma }}}^{-}+\zeta )|\tau |}]$, as shown in Fig. 3(a), where ${{\rm{\Gamma }}}^{\pm }={{\rm{\Gamma }}}_{{{\rm{S}}}_{2}/a{S}_{2}}^{\pm }$ and the parameter *ζ* represents the bandwidth of the source laser and is constant. By contrast, the correlation shapes obtained from the coherent and Kerr processes together are sharp and described by ${A}_{c}{=R}_{1}{|{{\rm{A}}}_{1}|}^{2}[{{\rm{e}}}^{-{2({\rm{\Gamma }}}_{1}^{+}{+{\rm{\Gamma }}}_{2}^{+}+\zeta )|{\rm{\tau }}|}{+e}^{-{2({\rm{\Gamma }}}_{1}^{-}{+{\rm{\Gamma }}}_{2}^{-}+\zeta )|{\rm{\tau }}|}-2\,\cos \,{({\rm{\Omega }}}_{{\rm{e}}}|{\rm{\tau }}|{)e}^{-{({\rm{\Gamma }}}_{1}^{+}{+{\rm{\Gamma }}}_{2}^{+}{+{\rm{\Gamma }}}_{1}^{-}{+{\rm{\Gamma }}}_{2}^{-}+\zeta )|{\rm{\tau }}|}]$, as shown in Fig. 3(a2) and (a3), where ${{\rm{\Gamma }}}_{1}^{\pm }={{\rm{\Gamma }}}_{{{\rm{S}}}_{2}/a{S}_{2}}^{\pm }$ and ${{\rm{\Gamma }}}_{2}={{\rm{\Gamma }}}_{FL}$.

The SP-FWM process dominates in Fig. 3(a1); according to the description given above, the correlation function shape is broad. For the cross-Kerr nonlinear processes shown in Fig. 3(a2) and (a3), because of the interactions between the hybrid FL and Stokes/anti-Stokes signals, the correlation function shape contains both sharp and broad peaks.

**Figure 4:**
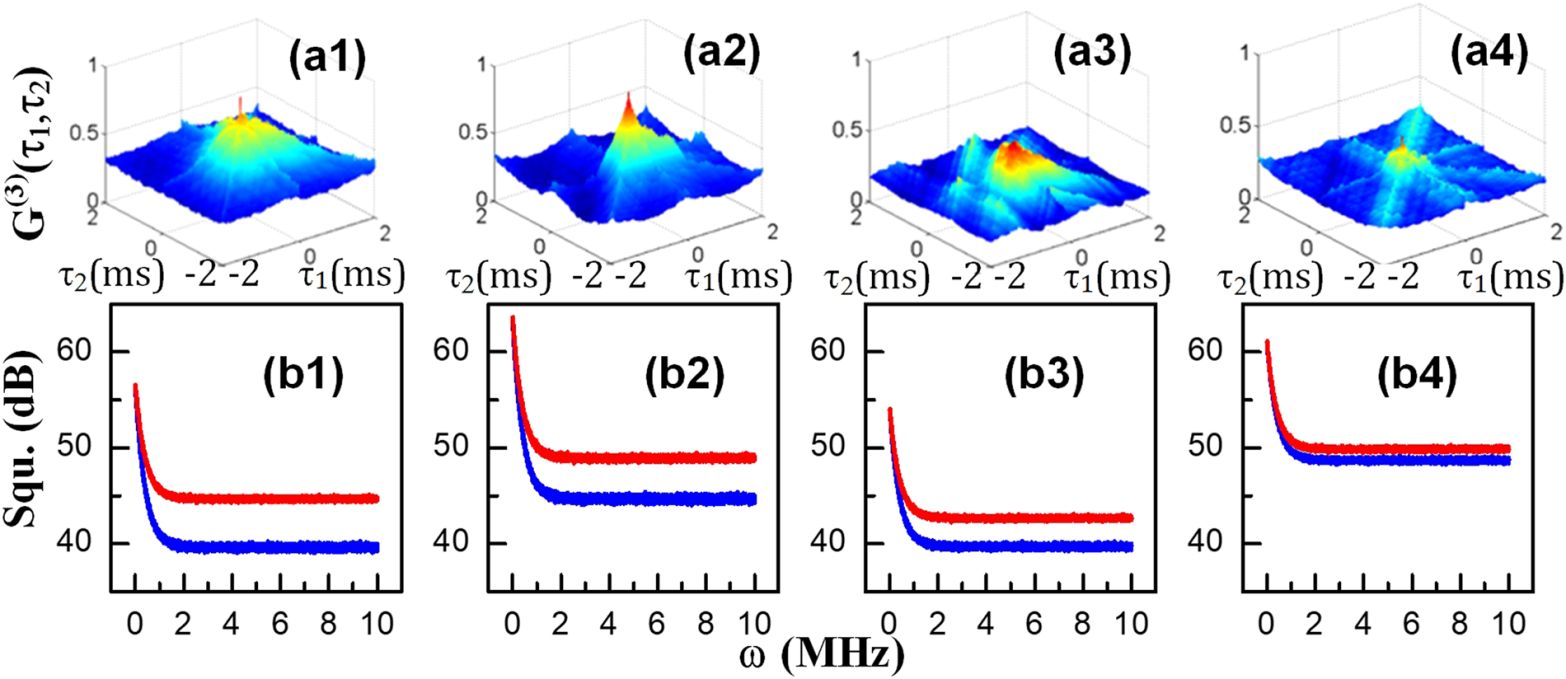
(**a1–a4**) Show the polarization dependence of the three-mode hybrid intensity-noise correlation of the Stokes and anti-Stokes SP-FWM and FL signals, and (**b1–b4**) show the corresponding intensity-difference squeezing as the polarization of *E*_2_ is changed from 0° to 15°, 30° and 45° while that of the pumping field *E*_1_ is kept linear.

**Figure 5:**
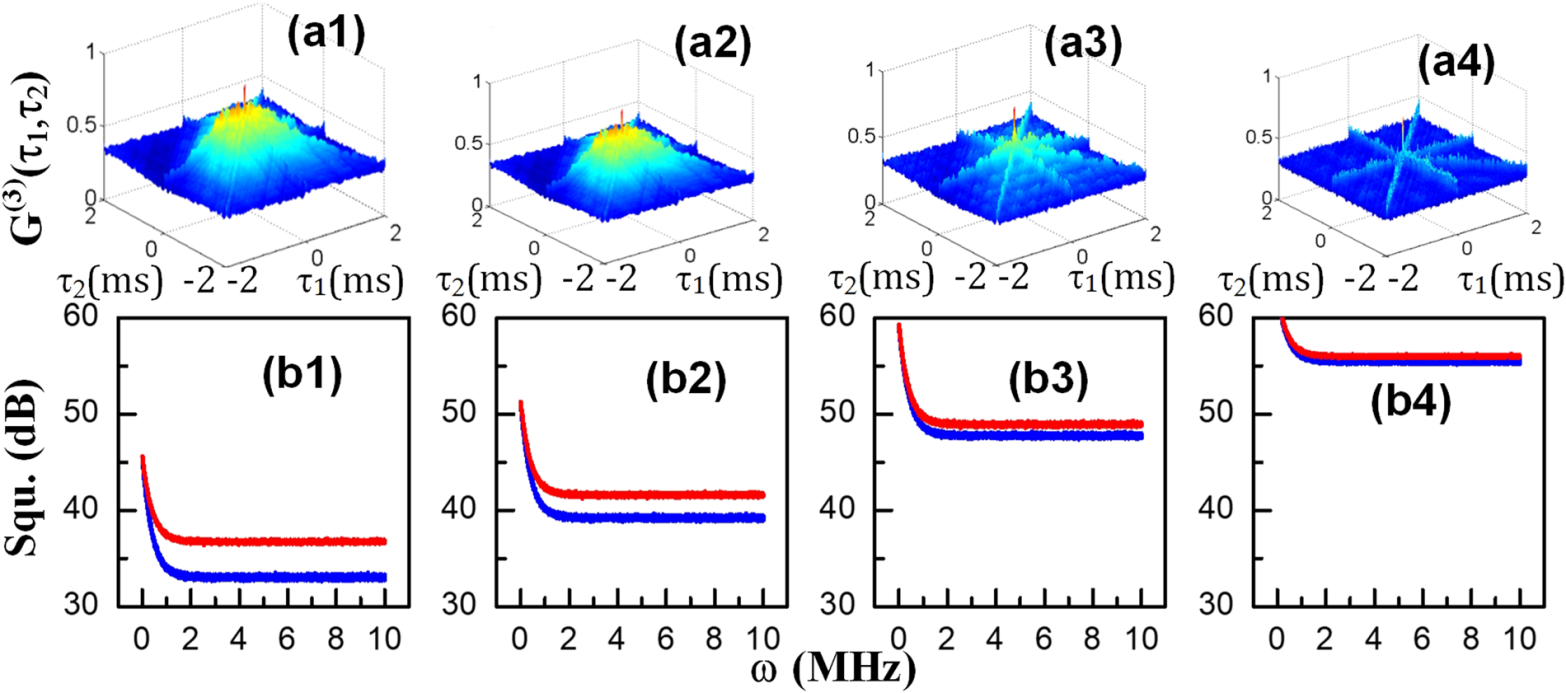
(**a1–a4**) show the power dependence of the three-mode hybrid intensity-noise correlation between the Stokes, anti-Stokes and FL signals; (**b1–b4**) show the corresponding intensity-difference squeezing as the power of *E*_2_ changes from high (6 mW, left) to low (1.5 mW, right) while *P*_1_ is kept constant.

In the following paragraphs, we will discuss the three-mode correlation and squeezing results of the three signals. In the Λ-type system shown in Fig. 1(a), the total intensity of the composite signal is ${\rho }_{comp}={\rho }_{20(S)}^{(3)}+{\rho }_{20({\rm{a}}S)}^{(3)}+{\rho }_{11(FL)}^{(4)}$. The intensity fluctuations $\delta {\hat{I}}_{1}({\tau }_{1})$, $\delta {\hat{I}}_{2}({\tau }_{2})$ and $\delta {\hat{I}}_{3}({\tau }_{3})$ were recorded by three detectors, and the triple-beam (S+aS+FL) intensity-noise correlations are shown in Fig. 4(a1–a4). Figures 5(a1,a2) and 6(a1–a3) were plotted by substituting these intensity-noise data into Eq. (21). Similarly, the corresponding three-mode intensity differences ${\delta }^{2}({\hat{I}}_{1}-{\hat{I}}_{2}-{\hat{I}}_{3})$ (blue curves in each panel) and noise sums ${\delta }^{2}({\hat{I}}_{1}+{\hat{I}}_{2}+{\hat{I}}_{3})$ (red curves in each panel) are shown in Figs 4(b1–b4), 5(b1,b2) and 6(b1–b3) were plotted by substituting these intensity fluctuations into Eq. (23).

**Figure 6:**
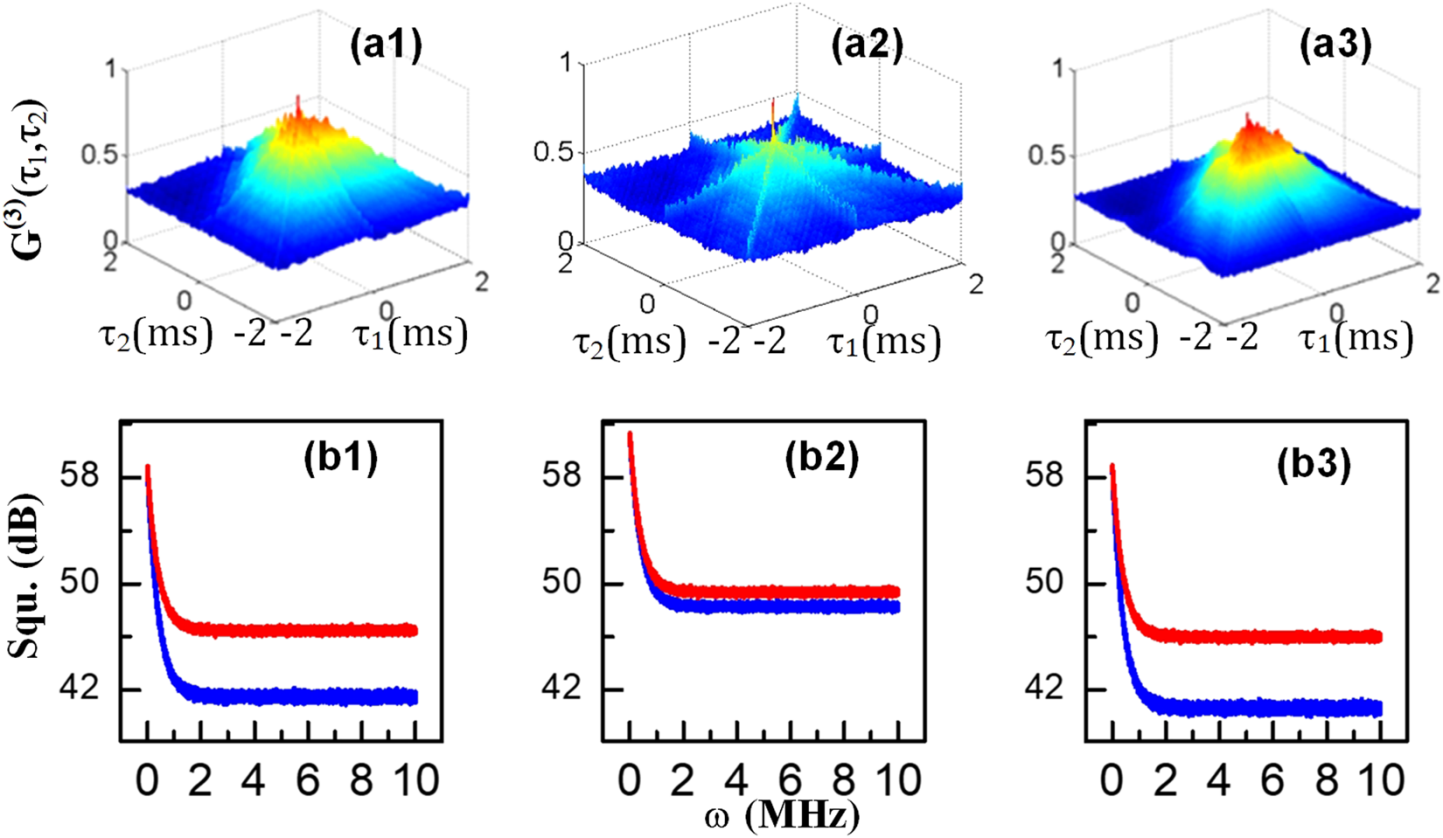
Variation in the hybrid three-mode intensity-noise correlation as the detuning changes. (**a1**–**a3**) Show ${G}^{(3)}({\tau }_{1},{\tau }_{2})$ versus the delay times (*τ*_1_, *τ*_2_) of the Stokes, anti-Stokes and fourth-order FL signals, and (**b1**–**b3**) show the corresponding three-mode intensity-difference squeezing as the detuning Δ_2_ is scanned from −200 GHz to +200 GHz for *θ* = 0°.

Here, we first study the differences in hybrid three-mode intensity-noise correlation and intensity-difference squeezing induced by the cross-Kerr effect with the dressing field in a linearly (*θ* = 0°) or circularly (*θ* = 45°) polarized state. Figure 4 shows the polarization dependence of the three-mode hybrid intensity-noise correlation when the power and detuning Δ_2_ of *E*_2_ are fixed to 6 mW and 0 GHz, respectively. Figure 4(a1–a4) shows the intensity-noise correlation between the Stokes, anti-Stokes and FL signals detected in the setup shown in Fig. 1(b). A QWP was inserted to control the polarization from linear to circular. The cross-Kerr effect changes with the polarization of the dressing field as given by Eqs (3)–(6).

Different degrees of correlation are observed for different polarization states, as shown in Fig. 4(a1–a4); this behaviour is determined by the Clebsch-Gordan coefficients and the polarization-induced dressing effects. The dressing effect of the field *E*_2_, which contains the terms ${c}_{x}^{2}{|{G}_{2}|}^{2}({\cos }^{4}\,\theta +{\sin }^{4}\,\theta )$ and ${c}_{y}^{2}{G}_{2}\,{\cos }^{2}\,\theta \,{\sin }^{2}\,\theta$, dominates over its gain effects, determined by ${c}_{x}^{2}{G}_{2}({\cos }^{4}\,\theta +{\sin }^{4}\,\theta )$ and ${c}_{y}^{2}{G}_{2}\,{\cos }^{2}\,\theta \,{\sin }^{2}\,\theta$, in Eqs (11) and (12); this effect causes the SP-FWM intensity to decrease as *θ* is changed from 0° (Fig. 4(a1)) to 15° (Fig. 4(a2)), 30° (Fig. 4(a3)) and, finally, to 45° (Fig. 4(a4)). In addition, the self-dressing terms of *E*_1_ (${|{G}_{1}|}^{2}/{{\rm{\Gamma }}}_{00}$, ${|{G}_{1}|}^{2}/{{\rm{\Gamma }}}_{22}$, ${|{G}_{1}|}^{2}/{{\rm{\Gamma }}}_{12}$, ${|{G}_{1}|}^{2}/{{\rm{d}}}_{00}$, ${|{G}_{1}|}^{2}/{{\rm{d}}}_{22}$, ${|{G}_{1}|}^{2}/{d}_{02}^{^{\prime} }$ and ${|{G}_{1}|}^{2}/{{\rm{d}}}_{02}$) in Eqs (11) and (12) cause the overall SP-FWM signal to decrease as the polarization changes from linear to circular. Similarly, the external dressing effect of the field *E*_2_, which contains the terms ${c}_{x}^{2}{|{G}_{2}|}^{2}({\cos }^{4}\,\theta +{\sin }^{4}\,\theta )$ and ${c}_{y}^{2}{G}_{2}\,{\cos }^{2}\,\theta \,{\sin }^{2}\,\theta$, dominates over the gain effect of the FL signal intensity (${\rho }_{20(FL)}^{(4)}$), determined by ${c}_{x}^{2}{|{G}_{1}|}^{2}{|{G}_{2}|}^{2}({\cos }^{4}\,\theta +{\sin }^{4}\,\theta )$, in Eq. (14). Thus, the three-mode hybrid intensity-noise correlation between the SP-FWM signals and the associated FL signals decreases in magnitude because of the dressing effect as shown in Fig. 4(a1–a4). Moreover, each field experiences a non-trivial phase shift as it propagates through the nonlinear medium. When a strong field passes through a Kerr medium, it causes the other output fields to undergo a phase shift. However, in our case, the nonlinear phase characterization (${n}_{0}+3{\chi }^{(3)}/2{n}_{0}{|E|}^{2}$) has a uniform impact of inducing a non-trivial phase on all outputs; this effect is why the correlation values are positive. Therefore, one can model this three-mode hybrid correlation as a three-mode hybrid entanglement of Stokes, anti-Stokes and FL photons in the form $|\psi (a)\rangle =(|001\rangle +|010\rangle +|100\rangle )/\sqrt{3}$. Because the amplitude of the correlation does not approach zero, one cannot treat this scenario as one of unentangled hybrid photons. Similarly, the three-mode squeezing between the intensity difference and the noise sum decreases from −4.59 dB (Fig. 4(b1)) to −1.28 dB (Fig. 4(b4)) and follows the same pattern as the corresponding correlation. These results are consistent with the theoretical formulations. In the treatment of the intensity-noise correlation, the three-mode entanglement criteria that determine the quantum correlation variances, described in Eq. (24), reduce to $\langle {\delta }^{2}({I}_{1}-{I}_{2})\rangle  < 1$, $\langle {\delta }^{2}({I}_{2}-{I}_{3})\rangle  < 1$ and $\langle {\delta }^{2}({I}_{3}-{I}_{1})\rangle  < 1$, with *I*_1_ = *X*_1_, *I*_2_ = *X*_2_ and *I*_3_ = *X*_3_, where the intensity fluctuations correspond to fluctuations of the amplitude quadratures and the corresponding phases are *Y*_1_ = *Y*_2_ = *Y*_3_ = 0 because of the uniformity of the Kerr effect on all outputs. The results presented in Fig. 4 clearly show the correlation between the two distinct states.

As the polarization of *E*_2_ changes from 0° to 45°, the anti-Stokes signal becomes small, as the Stokes and anti-Stokes signals are produced and determined by each other and the correlation between them is still strong. However, the amount of FL in the hybrid signal that is created by the Stokes and anti-Stokes signals will be reduced; hence, the degree of correlation between the Stokes/anti-Stokes and hybrid FL signals will decrease as the polarization of *E*_2_ changes from 0° to 45°. This effect will induce three-mode correlation changes, as shown in Fig. 5(a1–a4). Because the classical photonic flux part of the SP-FWM+FL signal has an equal role in the three-mode entanglement, the entanglement becomes a three-mode hybrid entanglement. The greater the FL intensity is, the greater is the hybrid entanglement. The shape of the three-mode correlation function is given by Eq. (22). Moreover, the degree of three-mode hybrid correlation and squeezing induced by the cross-Kerr effect decreases as the dressing field changes from being linearly polarized to being circularly polarized.

Figure 5 shows the three-mode hybrid intensity-noise correlation and intensity-difference squeezing of the Stokes, anti-Stokes and hybrid FL composite signals under the linearly polarized cross-Kerr effect as the power decreases from high (6 mW, left) to low (1.5 mW, right) while the polarization and detuning of *E*_2_ remain fixed at 0° and 0 GHz, respectively. Figure 5(a1–a4) show the three-mode intensity-noise correlation of the SP-FWM and hybrid FL signals, which decreases as the power decreases. This result is explained by the decrease in the total intensity of the hybrid signals as power of the dressing field decreases. When the power decreases, the external dressing terms ${|{G}_{2}|}^{2}/{{\rm{d}}}_{10}$, ${|{G}_{2}|}^{2}/{{\rm{d}}}_{20}$, and ${|{G}_{2}|}^{2}/{{\rm{\Gamma }}}_{10}$ and the self-dressing terms for the field *E*_1_ in Eqs (7) and (8) suppress the SP-FWM signal, which is consistent with what we observe in Fig. 1(d). In Fig. 1(d), curves (d1) and (d2) correspond to low and high powers, respectively. However, the gain effect of *E*_2_, determined by ${|{G}_{1}|}^{2}{|{G}_{2}|}^{2}$, dominates over its dressing effect, determined by ${|{G}_{2}|}^{2}/{d}_{10}$, ${|{G}_{2}|}^{2}/{d}_{20}$, and ${|{G}_{2}|}^{2}/{{\rm{\Gamma }}}_{10}$, in Eqs (7) and (8). Thus, at low power, the hybrid FL signal intensity dominates, and at high power, the SP-FWM signal intensity dominates. Therefore, in the correlation function, the magnitude of the broad shape becomes small and the magnitude of the sharp shape becomes large as the power of *E*_2_ decreases. The theoretical formulations agree well with the intensity distributions shown in Fig. 1(c) and (d) for hybrid signals and pure SP-FWM signals, respectively. Because the overall intensity decreases at low power, the correlation peaks also decrease from left to right, as shown in Fig. 5(a1–a4). Figure 5(b1–b4) show that the corresponding squeezing between the intensity difference and the noise sum decreases from −3.63 dB (Fig. 5(b1)) to −0.64 dB (Fig. 5(b4)).

In Fig. 6, we present the degree of correlation and squeezing of the composite signals under the linearly polarized cross-Kerr effect as the frequency detuning Δ_2_ changes from −200 GHz to +200 GHz while the power and polarization of *E*_2_ remain fixed at 6 mW and 0°, respectively. The three-mode signals from PMT1, PMT2 and PMT3 (Fig. 1(b)) were simultaneously recorded. Under off-resonance conditions (large detuning, Δ_2_), the detuning terms (${d}_{10}={{\rm{\Gamma }}}_{10}+i({{\rm{\Delta }}}_{1}-{{\rm{\Delta }}}_{2})$, ${d}_{20}={{\rm{\Gamma }}}_{20}+i{{\rm{\Delta }}}_{2}$) in Eqs (7) and (8) enhance ${\rho }_{20(S)}^{(3)}$ and ${\rho }_{20(aS)}^{(3)}$. Similarly, the terms appearing in Eq. (13), ${d}_{21}={{\rm{\Gamma }}}_{21}+i{{\rm{\Delta }}}_{2}$ and ${d}_{12}={{\rm{\Gamma }}}_{12}-i{{\rm{\Delta }}}_{2}$, enhance the emission of the hybrid FL signal at off-resonance points and suppress it at resonance (zero-detuning) points. This result is consistent with the results of ref. 29, in which the FL signal was found to be more sensitive to dressing than the SP-FWM signal. Thus, the squeezings between the intensity difference and noise sum in Fig. 6(a1) and (a3) are −5.41 dB and −5.68 dB, respectively, and are much larger than that in Fig. 6(a2) (−1.09 dB). Meanwhile, the detuning terms at off-resonance points in Eqs (3)–(6) enhance ${\rho }_{20(S-FL)}^{(3)}$, ${\rho }_{21(FL-S)}^{(3)}$, ${\rho }_{21(aS-FL)}^{(3)}$ and ${\rho }_{20(FL-aS)}^{(3)}$, respectively. Therefore, the cross-Kerr effect is enhanced under off-resonance conditions for a linearly polarized dressing field. Consequently, the magnitude of the broad shape in the correlation function is smaller at resonance points and larger at off-resonance points, whereas the magnitude of the sharp shape is larger at resonance points and smaller at off-resonance points. Because of the interactions between the hybrid FL signal and the Stokes/anti-Stokes signals, the correlations between the hybrid FL signal and the Stokes/anti-Stokes signals are lower at resonance points. Thus, the degree of hybrid intensity-noise correlation and intensity-difference squeezing can be controlled by controlling the amount of FL emission via the dressing field.

In conclusion, the two- and three-mode hybrid intensity-noise correlation and intensity-difference squeezing of SP-FWM and FL signals was studied by selectively controlling the polarization, power and frequency detuning of the input dressing laser in a Λ-type three-level atomic system in a Pr^3+^:Y_2_SiO_5_ crystal under the polarized cross-Kerr effect. The degree of three-mode hybrid correlation and squeezing degree induced by the cross-Kerr effect was found to decrease as the dressing field was changed from being linearly polarized to being circularly polarized. It was found that the degree of hybrid intensity-noise correlation and intensity-difference squeezing can be controlled by controlling the amount of FL emission from two Kerr processes. In particular, the two-mode correlation obtained from the interaction of the SP-FWM and Kerr processes is lower than that of the maximally entangled Stokes/anti-Stokes state. The variations in magnitude of the two- and three-mode intensity-noise correlation and intensity-difference squeezing are consistent with each other. Such a three-mode hybrid signal may have potential applications in long-distance communication, dense coding, all-optical communication and quantum storage on photonic chips.

## Methods

### Theoretical model

In this experiment, the correlation was calculated from the intensities of the signals obtained from three processes: coherent signals were obtained from the SP-FWM process, and non-coherent (hybrid) signals were obtained from two cross-Kerr nonlinear processes. The SP-FWM process produces Stokes and anti-Stokes signals. The two cross-Kerr nonlinear processes produce two hybrid FL+Stokes and FL+anti-Stokes signals via the interaction of the FL with the Stokes and anti-Stokes photons, respectively.

To generate the hybrid signal, we used a scheme similar to the one explained in ref. 5, where the cross-Kerr nonlinearity is responsible for the interaction of the SP-FWM and FL signals in the nonlinear crystal.

Cross-Kerr nonlinearity is essential for the generation of an entangled state. The Kerr nonlinearity is characterized by a refractive index of *n*_0_ + *n*_2_|*E*|^2^, where *n*_0_ is the weak-field linear refractive index term and *n*_2_ = 3*χ* ^(3)^/2*n*_0_ is a nonlinear refractive index term that is proportional to the field strength |*E*|^2^. Finally, the nonlinear refractive index can be characterized as *n*_0_ + 3*χ* ^(3)^/2*n*_0_|*E*|^2^.

Because of the coherence of the nonlinear medium, the interaction between the SP-FWM and FL outputs results in a hybrid signal (SP-FWM+FL), whose propagation can be described as follows:

1a $$c\frac{\partial {E}_{S}^{\ast }}{\partial z}={n}_{2}^{S-FL}{|{E}_{1}|}^{2}{E}_{FL}$$

1b $$c\frac{\partial {E}_{FL}}{\partial z}={n}_{2}^{FL-S}{|{E}_{1}|}^{2}{E}_{S}$$

2a $$c\frac{\partial {E}_{aS}^{\ast }}{\partial z}={n}_{2}^{aS-FL}{|{E}_{2}|}^{2}{E}_{FL}$$

2b $$c\frac{\partial {E}_{FL}}{\partial z}={n}_{2}^{FL-aS}{|{E}_{2}|}^{2}{E}_{aS}$$

where ${n}_{2}^{i}\propto {\chi }_{i}^{(3)}\propto {\rho }_{(i)}^{(3)}$ (*i* represents *S*-*FL*, *FL*-*S*, *aS*-*FL* or *FL*-*aS*). We can write the density matrix${\rho }_{(i)}^{(3)}$ as

3 $${\rho }_{20(S-FL)}^{(3)}=\frac{-i{G}_{S}{|{G}_{1}|}^{2}}{{({{\rm{\Gamma }}}_{20}+i{{\rm{\Delta }}}_{2})}^{2}({{\rm{\Gamma }}}_{00}+i{{\rm{\Delta }}}_{2}-i{{\rm{\Delta }}}_{1})}$$

4 $${\rho }_{21(FL-S)}^{(3)}=\frac{-i{G}_{aS}{|{G}_{1}|}^{2}}{{({{\rm{\Gamma }}}_{21}+i{{\rm{\Delta }}}_{1})}^{2}{{\rm{\Gamma }}}_{01}}$$

5 $${\rho }_{21(aS-FL)}^{(3)}=\frac{-i{G}_{aS}{|{G}_{2}|}^{2}[{c}_{x}^{2}({\cos }^{4}\,\theta +{\sin }^{4}\,\theta )+{c}_{y}^{2}\,{\cos }^{2}\,\theta \,{\sin }^{2}\,\theta ]}{{({{\rm{\Gamma }}}_{21}+i{{\rm{\Delta }}}_{1})}^{2}({{\rm{\Gamma }}}_{11}+i{{\rm{\Delta }}}_{1}-i{{\rm{\Delta }}}_{2})}$$

6 $${\rho }_{20(FL-aS)}^{(3)}=\frac{-i{G}_{S}{|{G}_{2}|}^{2}[{c}_{x}^{2}({\cos }^{4}\,\theta +{\sin }^{4}\,\theta )+{c}_{y}^{2}\,{\cos }^{2}\,\theta \,{\sin }^{2}\,\theta ]}{{({{\rm{\Gamma }}}_{20}+i{{\rm{\Delta }}}_{2})}^{2}{{\rm{\Gamma }}}_{10}}$$

where *θ* is the rotation angle of the QWP’s axis from the *x* axis, the *c*_*x*,*y*_ are the anisotropy factors in different directions in the crystal, ${{\rm{\Gamma }}}_{ij}$ is the transverse decay rate, and |*G*_*i*_|^2^ is the Rabi frequency of the field *E*_*i*._

The field intensities of these hybrid signals are given by ${I}_{S+FL}(z)=\langle {E}_{S}^{\ast }{E}_{S}\rangle$ and ${I}_{aS+FL}(z)=\langle {E}_{aS}^{\ast }{E}_{aS}\rangle$. Equations (1) and (2) describe two cross-Kerr nonlinear processes via the interaction of the FL with the Stokes and anti-Stokes signals, respectively. The first hybrid signal is obtain from the interaction of the Stokes and FL signals (Eq. (1a)), and conversely, the hybrid FL signal may produce the Stokes signal (Eq. (1b)). The second hybrid signal is obtained from the interaction of the anti-Stokes and FL signals (Eq. (2a)); similarly, this hybrid FL signal may produce the anti-Stokes signal (Eq. (2b)). Such Kerr nonlinearity is achieved under a strong magnitude of the pumping flux, which creates an interaction between the coherent SP-FWM and FL signals. The combination of a common energy level, a strong pumping field, a dressing field and propagation through a common medium creates such an interaction between the generated signals and the FL. This effect also allows one to create strongly correlated states^32, 33^.

In our scheme, we have demonstrated the correlation and squeezing of a hybrid signal consisting of Stokes, anti-Stokes and FL signals. In our mathematical model, we first adopt *pure* perturbation theory to obtain the Stokes and anti-Stokes signals under the weak-field approximation. Then, we incorporate dressing perturbation theory (DPT), in which the dressing terms of a strong field are included. In DPT, the density matrix elements of the Stokes and anti-Stokes signals and the associated FL signals are derived using the Liouville equation $\partial \hat{\rho }(t)/\partial t=(\,-\,i/\hslash )[\hat{H},\hat{\rho }(t)]-{\rm{\Gamma }}\hat{\rho }$, where $\hat{H}=({\hat{a}}_{S}^{\dagger }{\hat{a}}_{aS}^{\dagger }+{\hat{a}}_{S}{\hat{a}}_{aS})g/v$ is the Hamiltonian; ${\hat{a}}_{S}^{\dagger }({\hat{a}}_{S})$ and ${\hat{a}}_{aS}^{\dagger }({\hat{a}}_{aS})$ are creation and annihilation operators acting on the Stokes and anti-Stokes signals, respectively; ***v*** is the group velocity; and *g* is the nonlinear gain. For a Λ-type three-level system with two strong pumping fields *E*_1_ and *E*_2_, when the self-dressing effect of *E*_1_ and the external-dressing effect of *E*_2_ are considered, the third-order nonlinear density matrix elements of *E*_*S*_ and *E*_*aS*_ obtained via the perturbation chains ${\rho }_{00}^{(0)}\mathop{\longrightarrow }\limits^{{\omega }_{1}}{\rho }_{20}^{(1)}\mathop{\longrightarrow }\limits^{-{\omega }_{aS}}{\rho }_{10}^{(2)}\mathop{\longrightarrow }\limits^{{\omega }_{2}}{\rho }_{20(S)}^{(3)}$ and ${\rho }_{10}^{(0)}\mathop{\longrightarrow }\limits^{{\omega }_{2}}{\rho }_{20}^{(1)}\mathop{\longrightarrow }\limits^{-{\omega }_{S}}{\rho }_{00}^{(2)}\mathop{\longrightarrow }\limits^{{\omega }_{1}}{\rho }_{20(aS)}^{(3)}$, respectively, are given by

7 $${\rho }_{20(S)}^{(3)}=\frac{-i{G}_{1}{G}_{aS}^{\ast }{G}_{2}}{({d}_{02}^{^{\prime} }+\frac{{|{G}_{1}|}^{2}}{{{\rm{\Gamma }}}_{00}}+\frac{{|{G}_{1}|}^{2}}{{{\rm{\Gamma }}}_{22}}+\frac{{|{G}_{2}|}^{2}}{{d}_{10}})({{\rm{\Gamma }}}_{10}+\frac{{|{G}_{1}|}^{2}}{{{\rm{\Gamma }}}_{12}}+\frac{{|{G}_{2}|}^{2}}{{d}_{20}})({d}_{20}+\frac{{|{G}_{1}|}^{2}}{{d}_{00}}+\frac{{|{G}_{1}|}^{2}}{{d}_{22}}+\frac{{|{G}_{2}|}^{2}}{{{\rm{\Gamma }}}_{10}})}$$

8 $${\rho }_{20(aS)}^{(3)}=\frac{-i{G}_{1}{G}_{S}^{\ast }{G}_{2}}{({d}_{20}+\frac{{|{G}_{1}|}^{2}}{{d}_{00}}+\frac{{|{G}_{1}|}^{2}}{{d}_{22}}+\frac{{|{G}_{2}|}^{2}}{{{\rm{\Gamma }}}_{10}})({{\rm{\Gamma }}}_{00}+\frac{{|{G}_{1}|}^{2}}{{d}_{02}^{^{\prime} }}+\frac{{|{G}_{1}|}^{2}}{{d}_{02}})({d}_{02}^{^{\prime} }+\frac{{|{G}_{1}|}^{2}}{{{\rm{\Gamma }}}_{00}}+\frac{{|{G}_{1}|}^{2}}{{{\rm{\Gamma }}}_{22}}+\frac{{|{G}_{2}|}^{2}}{{d}_{10}})}$$

where ${d}_{02}^{^{\prime} }={{\rm{\Gamma }}}_{20}+i{{\rm{\Delta }}}_{1}$, ${d}_{10}={{\rm{\Gamma }}}_{10}+i({{\rm{\Delta }}}_{1}-{{\rm{\Delta }}}_{2})$, ${d}_{20}={{\rm{\Gamma }}}_{20}+i{{\rm{\Delta }}}_{2}$, ${d}_{00}={{\rm{\Gamma }}}_{00}+i({{\rm{\Delta }}}_{2}-{{\rm{\Delta }}}_{1})$, ${d}_{22}={{\rm{\Gamma }}}_{22}+i({{\rm{\Delta }}}_{2}-{{\rm{\Delta }}}_{1})$, ${d}_{02}={{\rm{\Gamma }}}_{02}-i{{\rm{\Delta }}}_{1}$, ${d}_{2}={{\rm{\Gamma }}}_{02}+i({{\rm{\Delta }}}_{2}-2{{\rm{\Delta }}}_{1})$, ${d}_{21}={{\rm{\Gamma }}}_{21}+i{{\rm{\Delta }}}_{2}$, and ${d}_{12}={{\rm{\Gamma }}}_{12}-i{{\rm{\Delta }}}_{2}$. When a QWP is inserted, it polarizes the light beam *E*_2_ into its components along the *x* and *y* axes. Thus, considering the different Clebsch-Gordan coefficients for various transitions, the Rabi frequency |*G*_2_|^2^ in Eqs (7) and (8) is replaced with ${c}_{x}^{2}{|{G}_{2}|}^{2}({\cos }^{4}\,\theta +{\sin }^{4}\,\theta )$ for ${\rho }_{(xxyy)}^{(3)}$ and ${c}_{y}^{2}{|{G}_{2}|}^{2}\,{\cos }^{2}\,\theta \,{\sin }^{2}\,\theta$ for ${\rho }_{(yyyy)}^{(3)}$, respectively, as related by the fourth-rank tensors in Eqs (9) and (10). Considering that the YSO crystal belongs to the ${C}_{2h}^{6}$ space point group, the non-vanishing tensor elements yield the effective susceptibility given by

9 $${\chi }_{eff}={\chi }_{xxyy}^{(3)}({\cos }^{4}\,\theta +{\sin }^{4}\,\theta )+2{\chi }_{yyyy}^{(3)}\,{\sin }^{2}\,\theta \,{\cos }^{2}\,\theta $$

Moreover, the third-order density matrix for the Stokes channel is

10 $$\begin{array}{rcl}{\rho }_{20(S)}^{(3)} & = & {\rho }_{20{(S)}^{xxyy}}^{(3)}+{\rho }_{20{(S)}^{yyyy}}^{(3)}\\ {\rho }_{20({\rm{a}}S)}^{(3)} & = & {\rho }_{20{({\rm{aS}})}^{xxyy}}^{(3)}+{\rho }_{20{({\rm{aS}})}^{yyyy}}^{(3)}\end{array}$$

Thus, in the dressed state picture, we obtain the third-order density matrices of the corresponding Stokes and anti-Stokes signals by substituting into Eq. (10) the new Rabi frequencies that appear in Eqs (7) and (8), as follows:

11 $$\begin{array}{rcl}{\rho }_{20(S)}^{(3)} & = & \frac{-\,i{c}_{x}^{2}({\cos }^{4}\,\theta +{\sin }^{4}\,\theta ){G}_{1}{G}_{aS}^{\ast }{G}_{2}}{[{d}_{02}^{^{\prime} }+\frac{{|{G}_{1}|}^{2}}{{{\rm{\Gamma }}}_{00}}+\frac{{|{G}_{1}|}^{2}}{{{\rm{\Gamma }}}_{22}}+\frac{{c}_{x}^{2}{|{G}_{2}|}^{2}({\cos }^{4}\,\theta +{\sin }^{4}\,\theta )}{{d}_{10}}]\times [{{\rm{\Gamma }}}_{10}+\frac{{|{G}_{1}|}^{2}}{{{\rm{\Gamma }}}_{12}}+\frac{{c}_{x}^{2}{|{G}_{2}|}^{2}({\cos }^{4}\,\theta +{\sin }^{4}\,\theta )}{{d}_{20}}]\times [{d}_{20}+\frac{{|{G}_{1}|}^{2}}{{d}_{00}}+\frac{{|{G}_{1}|}^{2}}{{d}_{22}}+\frac{{c}_{x}^{2}{|{G}_{2}|}^{2}({\cos }^{4}\,\theta +{\sin }^{4}\,\theta )}{{{\rm{\Gamma }}}_{10}}]}\\  &  & +\frac{-i{c}_{y}^{2}{\cos }^{2}\theta {\sin }^{2}\theta {G}_{1}{G}_{aS}^{\ast }{G}_{2}}{({d}_{02}^{^{\prime} }+\frac{{|{G}_{1}|}^{2}}{{{\rm{\Gamma }}}_{00}}+\frac{{|{G}_{1}|}^{2}}{{{\rm{\Gamma }}}_{22}}+\frac{{c}_{y}^{2}{|{G}_{2}|}^{2}\,{\cos }^{2}\,\theta \,{\sin }^{2}\,\theta }{{d}_{10}})\times ({{\rm{\Gamma }}}_{10}+\frac{{|{G}_{1}|}^{2}}{{{\rm{\Gamma }}}_{12}}+\frac{{c}_{y}^{2}{|{G}_{2}|}^{2}\,{\cos }^{2}\,\theta \,{\sin }^{2}\,\theta }{{d}_{20}})\,\times ({d}_{20}+\frac{{|{G}_{1}|}^{2}}{{d}_{00}}+\frac{{|{G}_{1}|}^{2}}{{d}_{22}}+\frac{{c}_{y}^{2}{|{G}_{2}|}^{2}\,{\cos }^{2}\,\theta \,{\sin }^{2}\,\theta }{{{\rm{\Gamma }}}_{10}})}\end{array}$$

and

12 $$\begin{array}{rcl}{\rho }_{20(aS)}^{(3)} & = & \frac{-i{c}_{x}^{2}({\cos }^{4}\,\theta +{\sin }^{4}\,\theta ){G}_{1}{G}_{S}^{\ast }{G}_{2}}{[{d}_{20}+\frac{{|{G}_{1}|}^{2}}{{d}_{00}}+\frac{{|{G}_{1}|}^{2}}{{d}_{22}}+\frac{{c}_{x}^{2}{|{G}_{2}|}^{2}({\cos }^{4}\,\theta +{\sin }^{4}\,\theta )}{{{\rm{\Gamma }}}_{10}}]\times ({{\rm{\Gamma }}}_{00}+\frac{{|{G}_{1}|}^{2}}{{d}_{02}^{\text{'}}}+\frac{{|{G}_{1}|}^{2}}{{d}_{02}})\times [{d}_{02}^{^{\prime} }+\frac{{|{G}_{1}|}^{2}}{{{\rm{\Gamma }}}_{00}}+\frac{{|{G}_{1}|}^{2}}{{{\rm{\Gamma }}}_{22}}+\frac{{c}_{x}^{2}{|{G}_{2}|}^{2}({\cos }^{4}\,\theta +{\sin }^{4}\,\theta )}{{d}_{10}}]}\\  &  & +\frac{-i{c}_{y}^{2}\,{\cos }^{2}\,\theta \,{\sin }^{2}\,\theta {G}_{1}{G}_{S}^{\ast }{G}_{2}}{({d}_{20}+\frac{{|{G}_{1}|}^{2}}{{d}_{00}}+\frac{{|{G}_{1}|}^{2}}{{d}_{22}}+\frac{{c}_{y}^{2}{|{G}_{2}|}^{2}\,{\cos }^{2}\,\theta \,{\sin }^{2}\,\theta }{{{\rm{\Gamma }}}_{10}})\times ({{\rm{\Gamma }}}_{00}+\frac{{|{G}_{1}|}^{2}}{{d}_{02}^{\text{'}}}+\frac{{|{G}_{1}|}^{2}}{{d}_{02}})\times ({d}_{02}^{^{\prime} }+\frac{{|{G}_{1}|}^{2}}{{{\rm{\Gamma }}}_{00}}+\frac{{|{G}_{1}|}^{2}}{{{\rm{\Gamma }}}_{22}}+\frac{{c}_{y}^{2}{|{G}_{2}|}^{2}\,{\cos }^{2}\,\theta \,{\sin }^{2}\,\theta }{{d}_{10}}),}\end{array}$$

respectively.

In the Λ-type three-level system shown in Fig. 1(a), with both the *E*_1_ and *E*_2_ fields open, the fourth-order FL signal is generated as depicted in Fig. 2. The intensity of the FL signal is obtained via the Liouville pathway ${\rho }_{10}^{(0)}\mathop{\longrightarrow }\limits^{{\omega }_{2}}{\rho }_{20}^{(1)}\mathop{\longrightarrow }\limits^{-{\omega }_{1}}{\rho }_{00}^{(2)}\mathop{\longrightarrow }\limits^{{\omega }_{1}}{\rho }_{20}^{(3)}\mathop{\longrightarrow }\limits^{-{\omega }_{2}}{\rho }_{11(FL)}^{(4)}$ and is given as

13 $${\rho }_{11(FL)}^{(4)}=\frac{{|{G}_{1}|}^{2}{|{G}_{2}|}^{2}}{{({d}_{20}+\frac{{|{G}_{1}|}^{2}}{{d}_{00}}+\frac{{|{G}_{1}|}^{2}}{{d}_{22}}+\frac{{|{G}_{2}|}^{2}}{{{\rm{\Gamma }}}_{10}})}^{2}({d}_{00}+\frac{{|{G}_{1}|}^{2}}{{d}_{20}}+\frac{{|{G}_{1}|}^{2}}{{d}_{2}})({{\rm{\Gamma }}}_{11}+\frac{{|{G}_{2}|}^{2}}{{d}_{21}}+\frac{{|{G}_{2}|}^{2}}{{d}_{12}})}$$

where $${d}_{20}={{\rm{\Gamma }}}_{20}+i{{\rm{\Delta }}}_{2}$$, $${d}_{00}={{\rm{\Gamma }}}_{00}+i({{\rm{\Delta }}}_{2}-{{\rm{\Delta }}}_{1})$$, $${d}_{22}={{\rm{\Gamma }}}_{22}+i({{\rm{\Delta }}}_{2}-{{\rm{\Delta }}}_{1})$$, $${d}_{2}={{\rm{\Gamma }}}_{02}+i({{\rm{\Delta }}}_{2}-2{{\rm{\Delta }}}_{1})$$, $${d}_{21}={{\rm{\Gamma }}}_{21}+i{{\rm{\Delta }}}_{2}$$, and $${d}_{12}={{\rm{\Gamma }}}_{12}-i{{\rm{\Delta }}}_{2}$$.

When dressing terms due to polarization changes of the field *E*_2_ are considered, the corresponding equation obtained by modifying Eq. (13) is

14 $$\begin{array}{rcl}{\rho }_{11(FL)}^{(4)} & = & \frac{{c}_{x}^{2}{|{G}_{1}|}^{2}{|{G}_{2}|}^{2}\,({\cos }^{4}\,\theta +{\sin }^{4}\,\theta )}{{[{d}_{20}+\frac{{|{G}_{1}|}^{2}}{{d}_{00}}+\frac{{|{G}_{1}|}^{2}}{{d}_{22}}+\frac{{c}_{x}^{2}{|{G}_{2}|}^{2}({\cos }^{4}\theta +{\sin }^{4}\theta )}{{{\rm{\Gamma }}}_{10}}]}^{2}\times ({d}_{00}+\frac{{|{G}_{1}|}^{2}}{{d}_{20}}+\frac{{|{G}_{1}|}^{2}}{{d}_{2}})\times [{{\rm{\Gamma }}}_{11}+\frac{{c}_{x}^{2}{|{G}_{2}|}^{2}({\cos }^{4}\,\theta +{\sin }^{4}\,\theta )}{{d}_{21}}+\frac{{c}_{y}^{2}{|{G}_{2}|}^{2}\,{\cos }^{2}\,\theta \,{\sin }^{2}\,\theta }{{d}_{12}}]}\\  &  & +\frac{{c}_{y}^{2}{|{G}_{1}|}^{2}{|{G}_{2}|}^{2}\,{\cos }^{2}\,\theta \,{\sin }^{2}\,\theta }{{({d}_{20}+\frac{{|{G}_{1}|}^{2}}{{d}_{00}}+\frac{{|{G}_{1}|}^{2}}{{d}_{22}}+\frac{{c}_{y}^{2}{|{G}_{2}|}^{2}{\cos }^{2}\theta {\sin }^{2}\theta }{{{\rm{\Gamma }}}_{10}})}^{2}\times ({d}_{00}+\frac{{|{G}_{1}|}^{2}}{{d}_{20}}+\frac{{|{G}_{1}|}^{2}}{{d}_{2}})\times ({{\rm{\Gamma }}}_{11}+\frac{{c}_{y}^{2}{|{G}_{2}|}^{2}\,{\cos }^{2}\,\theta \,{\sin }^{2}\,\theta }{{d}_{21}}+\frac{{c}_{y}^{2}{|{G}_{2}|}^{2}\,{\cos }^{2}\,\theta \,{\sin }^{2}\,\theta }{{d}_{12}})}\end{array}$$

For two weak multimode fields *E*_*S*_(*t*, *z*) and *E*_*aS*_(*t*, *z*), under the dressing conditions of the driving fields *E*_1_ and *E*_2_ and the light-matter interaction induced by the finite pulses shown in Fig. 1(a) in a localized medium, the propagation equations in the Heisenberg picture are given by refs 26, 27 $(\partial /\partial z+c\partial /\partial z){E}_{S}^{\ast }={\kappa }_{s}{E}_{1}{E}_{2}^{\ast }{E}_{aS}$ and $(\partial /\partial z+c\partial /\partial z){E}_{aS}^{\ast }={\kappa }_{aS}{E}_{1}{E}_{2}^{\ast }{E}_{S}$. This pair of equations describes the SP-FWM process. The Stokes and anti-Stokes signals are thus created and determined by each other.

These three nonlinear processes, the SP-FWM process and the two cross-Kerr nonlinear processes, create the hybrid FL state; hence, the degree of hybrid intensity-noise correlation and intensity-difference squeezing can be controlled by controlling the amount of FL in the hybrid signal.

The generation of Stokes and anti-Stokes fields via the SP-FWM process is illustrated in Fig. 1(a). The measured numbers of photons at detectors PMT1 and PMT2 for the anti-Stokes and Stokes channels, respectively, can be expressed as ref. 34

15 $$\begin{array}{rcl}{\hat{N}}_{aS} & = & {\hat{a}}_{aS}^{+}(L){\hat{a}}_{aS}(L)\\  & = & Q{\hat{a}}_{aS}^{+}(0){\hat{a}}_{aS}(0)+(Q-1){\hat{a}}_{S}(0){\hat{a}}_{S}^{+}(0)\\  &  & +\sqrt{Q(Q-1)}{\hat{a}}_{aS}^{+}(0){\hat{a}}_{S}^{+}(0)+\sqrt{Q(Q-1)}{\hat{a}}_{S}(0){\hat{a}}_{aS}(0)\end{array}$$

16 $$\begin{array}{rcl}{\hat{N}}_{S} & = & {\hat{a}}_{S}^{+}(L){\hat{a}}_{S}(L)\\  & = & Q{\hat{a}}_{S}^{+}(0){\hat{a}}_{S}(0)+(Q-1){\hat{a}}_{aS}(0){\hat{a}}_{aS}^{+}(0)\\  &  & +\sqrt{Q(Q-1)}{\hat{a}}_{S}^{+}(0){\hat{a}}_{aS}^{+}(0)+\sqrt{Q(Q-1)}{\hat{a}}_{aS}(0){\hat{a}}_{S}(0)\end{array}$$

where ${\hat{a}}_{S/{\rm{a}}S}^{+}({\hat{a}}_{S/{\rm{a}}S})$ is the creation (annihilation) operator that acts on the electromagnetic excitation of the Stokes (anti-Stokes) channel and *Q* = cosh^2^(*κL*) is the gain coefficient. The intensity of the signal (${I}_{j}({t}_{j})={N}_{j}{e}^{-{\Gamma }_{j}{t}_{j}}$) depends on the number of output photons, which in turn depends on the nonlinear susceptibility ${\chi }_{S,aS}^{(3)}$ and the pumping field amplitudes as follows: ${\chi }_{S,aS}^{(3)}=(N{\mu }_{S,aS}{\rho }_{S,aS}^{(3)})/({\varepsilon }_{0}{E}_{1}{E}_{2}{E}_{S,{\rm{a}}S})$, where the ${N}_{j}=\langle {\hat{{\rm{a}}}}_{j}^{+}{\hat{{\rm{a}}}}_{j}\rangle$ are the numbers of photons in Eqs (15) and (16) and the *μ*_*S*,*aS*_ are the dipole moments.

Because of the relationship ${\kappa }_{S/aS}\propto {\chi }_{S/aS}^{(3)}\propto {\rho }_{S/aS}^{(3)}$, the output signals from the FWM process can be modified by applying a dressing field and controlling the power, frequency detuning and polarization of that dressing field. Below, the results for three-mode hybrid intensity-noise correlation and intensity-difference squeezing are analysed.

In the two-mode case, the intensity-noise correlation from the coherent process is given by

17 $${G}_{S-aS}^{(2)}=\frac{\langle \delta {\hat{I}}_{S}({t}_{S})\delta {\hat{I}}_{aS}({t}_{aS})\rangle }{\sqrt{\langle \delta {\hat{I}}_{S}{({t}_{S})}^{2}\rangle \langle \delta {\hat{I}}_{aS}{({t}_{aS})}^{2}\rangle }}.$$

For this two-mode correlation, the degree of intensity-difference squeezing from the coherent SP-FWM process is given by ref. 35

18 $$S{q}_{S-aS}^{(2)}={\rm{L}}{\rm{o}}{{\rm{g}}}_{10}\frac{\langle {\delta }^{2}({\hat{I}}_{S}-{\hat{I}}_{aS})\rangle }{\langle {\delta }^{2}({\hat{I}}_{S}+{\hat{I}}_{aS})\rangle }$$

where $\langle {\delta }^{2}({\hat{I}}_{S}-{\hat{I}}_{aS})\rangle$ is the mean square deviation of the intensity difference and $\langle {\delta }^{2}({\hat{I}}_{S}+{\hat{I}}_{aS})\rangle$ is the mean square deviation of the noise sum.

Similarly, for the two cross-Kerr nonlinear processes illustrated in Fig. 2 (see Eqs (1) and (2)), each involving one signal from the SP-FWM process, the two-mode hybrid correlations are given by

19 $${G}_{S/aS-FL}^{(2)}(\tau )=\frac{\langle \delta {\hat{I}}_{S/aS}({t}_{S/aS})\delta {\hat{I}}_{FL}({t}_{FL})\rangle }{\sqrt{\langle \delta {\hat{I}}_{S/aS}{({t}_{S/aS})}^{2}\rangle \langle \delta {\hat{I}}_{FL}{({t}_{FL})}^{2}\rangle }}$$

Meanwhile, the corresponding degrees of two-mode hybrid intensity-difference squeezing from the coherent and Kerr processes together are given by

20 $$S{q}_{S/aS-FL}^{(2)}={\rm{L}}{\rm{o}}{{\rm{g}}}_{10}\frac{\langle {\delta }^{2}({\hat{I}}_{S/aS}-{\hat{I}}_{FL})\rangle }{\langle {\delta }^{2}({\hat{I}}_{S/aS}+{\hat{I}}_{FL})\rangle }$$

where $\langle {\delta }^{2}({\hat{I}}_{S/aS}-{\hat{I}}_{FL})\rangle$ is the mean square deviation of the intensity difference and $\langle {\delta }^{2}({\hat{I}}_{S/aS}+{\hat{I}}_{FL})\rangle$ is the mean square deviation of the noise sum.

The three-mode correlation of the intensity fluctuations for the three optical beams ${G}^{(3)}({\tau }_{1},{\tau }_{2},{\tau }_{3})$ obtained from the SP-FWM process and the cross-Kerr nonlinear processes as a function of the time delay *τ* is given by

21 $${G}^{(3)}({\tau }_{1},{\tau }_{2},{\tau }_{3})=\frac{\langle (\delta {\hat{I}}_{1}({\tau }_{1}))(\delta {\hat{I}}_{2}({\tau }_{2}))(\delta {\hat{I}}_{3}({\tau }_{3}))\rangle }{\sqrt{\langle {(\delta {\hat{I}}_{1}({\tau }_{1}))}^{2}\rangle \langle {(\delta {\hat{I}}_{2}({\tau }_{2}))}^{2}\rangle \langle {(\delta {\hat{I}}_{3}({\tau }_{3}))}^{2}\rangle }}$$

The shape of the three-mode correlation function is described as

22 $$A=B\int d\,{\omega }_{2}{|{e}^{-i{\omega }_{2}{\tau }_{3}}\frac{{\kappa }_{1}{\kappa }_{2}{\sinh }^{2}({\rm{\Omega }}L)\cosh ({\rm{\Omega }}t)}{{{\rm{\Omega }}}^{2}}|}^{2}-C\int d\,{\omega }_{2}[{e}^{-i{\omega }_{2}({\tau }_{2}-{\tau }_{1})}\frac{{\kappa }_{1}\,\sinh ({\rm{\Omega }}L)\,\cosh ({\rm{\Omega }}L)}{{\rm{\Omega }}}]$$

where B and C are parameters that affect the magnitude of the correlation, ${\rm{\Omega }}$ is the Rabi frequency, *L* is the length of the medium, and *κ* is the nonlinear coefficient. The function given in Eq. (22) is related to *τ* and, as a result, determines the shape of the three-mode correlation function ${G}^{(3)}({\tau }_{1,}{\tau }_{2,}{\tau }_{3})$.

In the three-mode case, the corresponding degree of intensity-difference squeezing is defined as ref. 19

23 $$S{q}^{(3)}={\rm{L}}{\rm{o}}{{\rm{g}}}_{10}\frac{\langle {\delta }^{2}({\hat{I}}_{1}-{\hat{I}}_{2}-{\hat{I}}_{3})\rangle }{\langle {\delta }^{2}({\hat{I}}_{1}+{\hat{I}}_{2}+{\hat{I}}_{3})\rangle }$$

where $$\langle {\delta }^{2}({\hat{I}}_{1}+{\hat{I}}_{2}+{\hat{I}}_{3})\rangle =\langle {({\hat{I}}_{1}+{\hat{I}}_{2}+{\hat{I}}_{3})}^{2}\rangle -{\langle {\hat{I}}_{1}+{\hat{I}}_{2}+{\hat{I}}_{3}\rangle }^{2}$$ and $${\delta }^{2}({\hat{I}}_{1}-{\hat{I}}_{2}-{\hat{I}}_{3})=\langle {({\hat{I}}_{1}-{\hat{I}}_{2}-{\hat{I}}_{3})}^{2}\rangle -$$ $${\langle {\hat{I}}_{1}-{\hat{I}}_{2}-{\hat{I}}_{3}\rangle }^{2}$$.

To study the correlations and squeezing of Stokes, anti-Stokes and FL signals transmitted through Pr^3+^:YSO, the dependence of the signal intensities on time, 〈*I*_*i*_〉 + *δI*_*i*_(*τ*) (i = aS, S, FL), was recorded using digital oscilloscopes and PMTs. Here, the 〈*I*_*i*_〉 are the average intensities of the anti-Stokes, Stokes and FL beams, and the *δI*_*i*_(*τ*) are the corresponding intensity fluctuations. Then, the intensity fluctuations were averaged by a fast gated integrator over 10 pulses. The temporal waveform correlations between the Stokes, anti-Stokes and FL signals were investigated based on Eqs (17), (19) and (21) under a variety of conditions. Additionally, using the intensity fluctuations recorded by the PMTs, the measured intensity fluctuations in the Stokes, anti-Stokes and FL channels were subtracted from and added to each other and were then analysed using a spectrum analyser to investigate the relative noise power between the intensity difference and the noise sum, as described by Eqs (18), (20) and (23).

Furthermore, the three-mode entanglement condition is given by

24 $$\begin{array}{l}\langle {\delta }^{2}({{\rm{X}}}_{2}-{{\rm{X}}}_{3})\rangle +\langle {\delta }^{2}({g}_{1}{Y}_{1}+{Y}_{2}+{Y}_{3})\rangle  < 1\\ \langle {\delta }^{2}({{\rm{X}}}_{3}-{{\rm{X}}}_{1})\rangle +\langle {\delta }^{2}({Y}_{1}+{g}_{2}{Y}_{2}+{Y}_{3})\rangle  < 1\\ \langle {\delta }^{2}({{\rm{X}}}_{1}-{{\rm{X}}}_{2})\rangle +\langle {\delta }^{2}({Y}_{1}+{Y}_{2}+{g}_{3}{Y}_{3})\rangle  < 1\end{array}$$

where *g*_1_, *g*_2_ and *g*_3_ are the coupling coefficients and *X*_1_, *X*_2_, and *X*_3_ and *Y*_1_, *Y*_2_, and *Y*_3_ are the intensities and phases, respectively, of the three corresponding signals.

## References

[1] Glauber RJ (1963). Coherent and incoherent states of the radiation field. Phys. Rev..

[2] Grangier P, Roger G, Aspect A (1986). Experimental Evidence for a Photon Anticorrelation Effect on a Beam Splitter: A New Light on Single-Photon Interferences. Europhys. Lett..

[3] Brown RH, Twiss RQ (1956). Correlation between Photons in two Coherent Beams of Light. Nature.

[4] Agarwal GS, Jha SS (1979). Antibunching effects in resonant raman scattering involving laser fields of arbitrary strengths. Zeitschrift für Phys. B.

[5] Gerry C (1999). Generation of optical macroscopic quantum superposition states via state reduction with a Mach-Zehnder interferometer containing a Kerr medium. Phys. Rev. A.

[6] Duan L-M, Lukin MD, Cirac JI, Zoller P (2001). Long-distance quantum communication with atomic ensembles and linear optics. Nature.

[7] Vered RZ, Shaked Y, Ben-Or Y, Rosenbluh M, Pe’Er A (2015). Classical-to-quantum transition with broadband four-wave mixing. Phys. Rev. Lett..

[8] Lee SW, Jeong H (2013). Near-deterministic quantum teleportation and resource-efficient quantum computation using linear optics and hybrid qubits. Phys. Rev. A.

[9] Jeong H (2014). Generation of hybrid entanglement of light. Nat. Photonics.

[10] Kwon H, Jeong H (2013). Violation of the Bell-Clauser-Horne-Shimony-Holt inequality using imperfect photodetectors with optical hybrid states. Phys. Rev. A.

[11] Park K, Lee SW, Jeong H (2012). Quantum teleportation between particlelike and fieldlike qubits using hybrid entanglement under decoherence effects. Phys. Rev. A.

[12] Ou Z, Lu Y (1999). Cavity Enhanced Spontaneous Parametric Down-Conversion for the Prolongation of Correlation Time between Conjugate Photons. Phys. Rev. Lett..

[13] Van Loock P (2006). Hybrid quantum repeater using bright coherent light. Phys. Rev. Lett..

[14] Mao D (2013). Flexible high-repetition-rate ultrafast fiber laser. Sci. Rep..

[15] Liu X (2005). Stable and uniform dual-wavelength erbium-doped fiber laser based on fiber Bragg gratings and photonic crystal fiber. Opt. Express.

[16] Liu X-M (2006). Four-wave mixing self-stability based on photonic crystal fiber and its applications on erbium-doped fiber lasers. Opt. Commun..

[17] Liu XM (2008). Theory and experiments for multiple four-wave-mixing processes with multifrequency pumps in optical fibers. Phys. Rev. A.

[18] Liu XM (2010). Broad and tunable multiwavelength fiber laser at the assistance of modulation-instability-assisted four-wave mixing. Laser Phys..

[19] Qin Z (2014). Experimental generation of multiple quantum correlated beams from hot rubidium vapor. Phys. Rev. Lett..

[20] Equall RW, Cone RL, MacFarlane RM (1995). Homogeneous broadening and hyperfine structure of optical transitions in Pr3+:Y2SiO5. Phys. Rev. B.

[21] Zhang Y, Khadka U, Anderson B, Xiao M (2009). Temporal and spatial interference between four-wave mixing and six-wave mixing channels. Phys. Rev. Lett..

[22] Zhang Y (2011). Four-wave mixing dipole soliton in laser-induced atomic gratings. Phys. Rev. Lett..

[23] Ding D-S (2015). Hybrid-cascaded generation of tripartite telecom photons using an atomic ensemble and a nonlinear waveguide. Optica.

[24] Zha X, Ahmed I, Zhang Y (2016). 3-Uniform states and orthogonal arrays. Results Phys..

[25] Abdisa G (2016). Controllable hybrid shape of correlation and squeezing. Phys. Rev. A.

[26] Lukin MD, Imamoglu A (2000). Nonlinear Optics and Quantum Entanglement of Ultra-Slow Single Photons. Phys. Rev. Lett..

[27] Gea-Banacloche J (2010). Impossibility of large phase shifts via the giant Kerr effect with single-photon wave packets. Phys. Rev. A.

[28] Li C (2015). Multi-dressing time delayed fourth- and sixth-order fluorescence processes in Pr^3+^:YSO. RSC Adv..

[29] Lan H (2015). Competition between spontaneous parametric four-wave mixing and fluorescence in Pr^3+^:YSO. Laser Phys. Lett..

[30] Ahmed I (2015). Dressed intensity noise correlation and intensity-difference squeezing of spontaneous parametric four-wave mixing process in a Pr^3+^:YSO crystal. Opt. Express.

[31] Zheng H (2013). Parametric amplification and cascaded-nonlinearity processes in common atomic system. Sci. Rep..

[32] Scully, M. O. & Zubairy, M. S. *Quantum optics*, doi:10.1017/CBO9780511813993 (Cambridge University Press, 1997).

[33] Shapiro JH, Razavi M (2007). New Journal of Physics. New J. Phys..

[34] Chen HX (2014). Parametric amplification of dressed multi-wave mixing in an atomic ensemble. Laser Phys. Lett..

[35] Boyer V, Marino AM, Pooser RC, Lett PD (2009). Entangling light in its spatial degrees of freedom with four-wave mixing in an atomic vapor. Chem. Phys. Chem.

